# The Use of Eye-Tracking Technology in Pediatric Orofacial Clefts: A Systematic Review and Meta-Analysis

**DOI:** 10.3390/children10081425

**Published:** 2023-08-21

**Authors:** Ghalia Y. Bhadila, Dana A. Alyafi

**Affiliations:** 1 Department of Pediatric Dentistry, Faculty of Dentistry, King Abdulaziz University, Jeddah 21589, Saudi Arabia; 2 Department of Orthodontics, Faculty of Dentistry, King Abdulaziz University, Jeddah 21589, Saudi Arabia; dalyafie@kau.edu.sa

**Keywords:** cleft lip, craniofacial anomalies, pediatric dentistry, eye tracking, systematic review

## Abstract

This systematic review and meta-analysis assessed the quality of the peer-reviewed literature and evaluated the usefulness of eye-tracking technology in evaluating observers’ perceptions of pediatric patients with orofacial clefts. PubMed, Science Direct, Wiley, and Web of Science were searched. Articles were screened in accordance with the Preferred Reporting Items for Systematic Review and Meta-analysis guidelines, and their methodological quality was assessed. Of the 10,254 identified studies, 12 were included. Eleven studies were cross-sectional, and one was a prospective cohort study. The main areas of interest analyzed were the eyes, nose, and mouth. Nine studies used assessment scales to analyze the link between perceived attractiveness and visualization patterns and measures. For the fixation duration outcome, six studies were eligible for inclusion in the meta-analysis. All studies reported on fixation duration in milliseconds and reported on a standard deviation. The meta-analysis demonstrated a significant difference in the measurements between the control groups and the patients with orofacial clefts. This might indicate the usefulness of eye-tracking technology as a metric for assessing the success of cleft repairs based on the perceptions of different populations. Future studies should be comprehensively reported on for comparability and reproducibility purposes.

## 1. Introduction

Cleft lip and/or palate (CL+/P) affects more than 10 million infants worldwide [1]. Approximately every 3 min, a child with some form of orofacial cleft is born [1]. Primary lip surgery is performed from infancy to 3 months of age to enhance esthetics and function [2]. This is followed by surgical correction of the palate by 6–9 months of age to allow for better dental and facial growth, feeding, and speech [3]. Once the upper canines begin their eruption path, alveolar bone grafting is needed at 7–9 years of age [4]. Repeated surgical interventions to enhance facial esthetic outcomes during the developmental years of these patients might result in secondary deformities [4].

A negative social perception is often associated with patients with CL+/P due to their perceived unattractive facial appearance [5]. Thus, numerous studies have recruited laypeople, professionals, and potential peers to assess their perceptions of the attractiveness of patients with CL+/P [6,7,8]. Furthermore, the facial perceptions of different population groups are essential because of their indirect effects on the emotional and social well-being of children with orofacial clefts [6,7,8].

The use of eye-tracking technology in dentistry is in its infancy. Eye-tracking machines allow for the tracing of gaze patterns, fixation duration (the time spent gazing on a certain region), fixation counts (number of eye visits on a single location), time to first fixation, and other parameters [9]. Recently, an increasing number of studies have utilized eye-tracking technology to assess observers’ perceptions of patients with CL+/P [7,10,11,12].

Several types of eye trackers are available commercially. Stationary eye-tracker machines can be used in laboratories, while mobile eye-tracker machines can be used by observers carrying out live interactions. The two most commonly used eye-tracker machines are primarily manufactured by Tobii (https://www.tobii.com/) (accessed on 13 August 2023) and SR Research (https://www.srresearch.com/) (accessed on 13 August 2023) [13].

While using eye trackers, the accuracy of obtaining measurements is increased when a chin rest is employed; this is important when there is a need to detect small eye movements. However, measurements that are recorded without stabilizing the head do generate readings with an acceptable level of accuracy [13].

Recording the visual perceptions of individuals using eye-tracking machines allows for objective measurements to be obtained. This can provide insights into the conscious and unconscious preferences of people and allows for the success of the esthetic corrective treatments of patients with CL+/− [14] to be assessed.

As this technology is in its initial application phase, it is important to evaluate and assess the quality of the studies reported so far, and to develop recommendations to ensure reproducibility for outcome comparisons. To the authors’ knowledge, no study has summarized the available literature on the utilization of eye-tracking technology in the assessment of observers’ perceptions of pediatric patients with orofacial clefts. Therefore, the purpose of this systematic review and meta-analysis is to assess the quality of the peer-reviewed literature and to evaluate the usefulness of eye-tracking technology in evaluating observers’ perceptions of pediatric patients with orofacial clefts.

## 2. Materials and Methods

### 2.1. Systematic Literature Search

This systematic review followed the criteria outlined in the Preferred Reporting Items for Systematic Reviews and Meta-Analysis (PRISMA) guidelines [15]. A comprehensive search of the following databases was performed to identify the relevant studies: PubMed, Science Direct, Wiley, and Web of Science. Each database was searched from inception to December 2022 using the specific keywords shown in Table 1. The Boolean operators (AND) and (OR) were used to narrow the search results. A hand search of the reference list of the included articles was also performed.

The population (P), intervention (I), comparison (C), outcome (O) elements were used to identify the studies in this systematic review:

P: Observers including laypeople, mothers, medical professionals, and other children;
I: Pediatric patients with orofacial clefts;
C: Non-cleft patients;
O: Visual perception parameters measured by eye trackers.

### 2.2. Inclusion and Exclusion Criteria

The studies included in this systematic review utilized eye-tracking technology to assess the observers’ visual perceptions of individuals with CL+/P. Only articles in the English language were included and the search was limited to studies conducted on pediatric populations (from birth to 18 years of age).

Review articles, abstracts, conference proceedings, editorials, comments, and book chapters were excluded from the search. Other exclusion criteria were clefts other than orofacial clefts; stimuli displaying participants older than 18 years of age; and studies that assessed visual perception toward participants with orofacial clefts without using an eye-tracking device.

### 2.3. Data Extraction and Synthesis

All the identified articles were imported into Covidence systematic review software (Veritas Health Innovation, Melbourne, Australia) to manage the screening process and eliminate duplicates. Two independent reviewers (G.B. and D.A.) completed the screening by title and abstract based on the inclusion and exclusion criteria. The selected articles were further assessed by a full-text review to determine eligibility. Disagreements between the authors were resolved by discussion until a consensus was reached.

The data were synthesized narratively and presented in structured tables with the primary outcomes. The extracted data included the study design; observer population characteristics; stimulus material standardization and defect displayed; eye-tracking application and system set-up; identified areas of interest (AOI); and measurement parameters. The assessment scales used, and their relevant results, were documented.

### 2.4. Quality Assessment

The methodological quality of the included studies was assessed by two independent reviewers (G.B. and D.A.) using the NIH Tool for Observational Cohort and Cross-Sectional Studies [16]. The level of agreement between the two authors was evaluated by Cohen’s Kappa value (Kappa = 0.83). In the case of any disagreement between the two authors, a discussion of the value was carried out until they reached a consensus. The items evaluated by the tool assessed the study for several biases, such as population selection, variable measurements, attrition, and the presence of confounding factors. The questions were answered and scored as follows: yes (1 point), no (0 points), or cannot determine, not applicable or not reported (0 points). An overall score was then calculated for each study to determine the risk-of-bias level. Studies that scored 14–10 were at a low risk of bias, 9–5 were at a moderate risk of bias and 4–0 were at a high risk of bias [17] (Appendix A).

### 2.5. Quantitative Data Analysis

For the quantitative data analysis of the study outcomes, we estimated the mean difference between experimental and control groups with a 95% confidence interval using a random effects meta-analysis. We pooled data across studies when the following three criteria were met: a minimum of three studies were sufficiently homogenous in outcome reporting (i.e., used the same measurement tool), reported results for an experimental and a control group, and had complete reporting of the outcome and its measure of variance. To determine the sample size of the experimental and control groups, we calculated the product of the number of participants by the number of images assessed in each group (Appendix B).

## 3. Results

**Figure 1:**
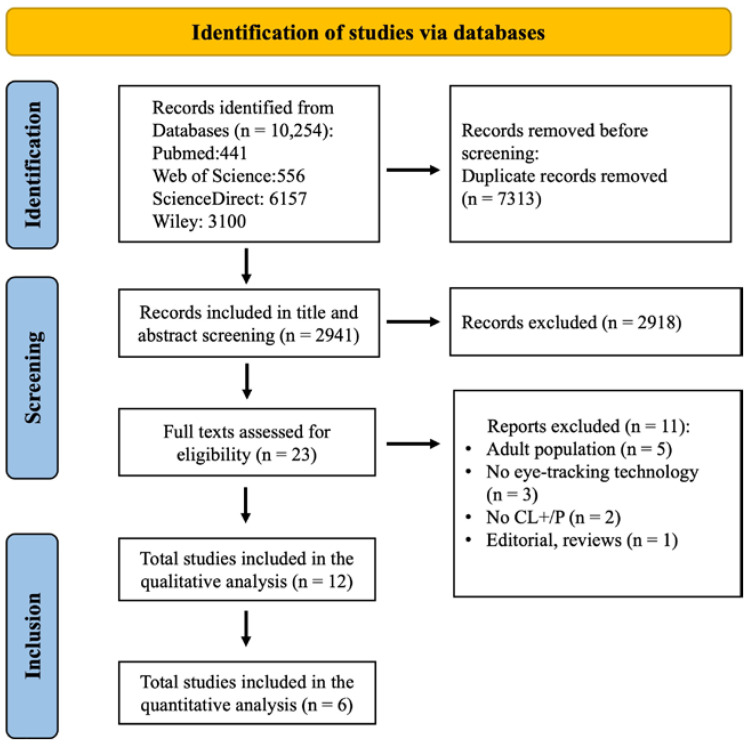
PRISMA flowchart diagram presenting the selection scheme for the articles.

In total, 10,254 studies were identified; however, 7313 duplicates were removed. As a result, only 2941 studies were screened by title and abstract. A total of 2918 irrelevant studies were excluded. The remaining 23 full-text studies were assessed for eligibility. Eleven studies were excluded because they did not fulfill the inclusion criteria. Thus, a total of 12 studies were included in the study [6,7,8,10,11,12,18,19,20,21,22,23] (Figure 1).

### 3.1. Study Design and Characteristics

The details of the study design of each included article are presented in Table 2. The studies were conducted on populations across 7 countries, primarily the United States (3 studies) [7,11,23], Brazil (3 studies) [6,19,20], Germany (2 studies) [8,12], the United Kingdom (2 studies) [10,22], and Canada (1 study) [21]. One study obtained results from a sample population from several countries (the United States, Egypt, and Thailand) [18]. All these studies were published in the past decade, from 2017 to 2022. Eleven studies were cross-sectional, and one was a prospective cohort study.

### 3.2. Observers and Stimulus Specifications

Table 3 lists the observers and stimulus material specifications. The observers in the study sample populations included laypeople, professionals with plastic surgery experience, children and adolescents, and mothers of infants with CL+/P. The sample size across all studies ranged from 11–403 participants (mean = 79.8), with an even sex distribution (female-to-male ratio 1:1.04). The participants’ visual acuities were determined by self-report in eight studies, one study tested participants’ vision [18], but three studies did not report on it [7,10,23].

The stimulus materials presented to the sample observers in all the included studies consisted of pediatric populations with ages ranging from infancy to adolescence. Eleven studies used static images that displayed unrepaired CL+/P defects, unilateral repaired CL+/P with or without a secondary defect, or bilateral CL+/P repair. One study investigated live interactions of mothers and their infants with CL+/P defects [10].

### 3.3. Eye-Tracking Apparatus and Settings

The eye-tracking apparatuses and applications used in all the included studies are detailed in Table 4. Various eye-tracking systems were used to record visual gaze. The most frequently used eye-tracking system was a screen-based machine (n = 9), followed by eye-tracker glasses (n = 2) [10,11], and a head-mounted eye tracker (n = 1) [22]. The calibration of observers to the eye-tracker system was implemented prior to performing the viewing task (n = 11). One study did not report on the calibration process. In most studies, the observers were seated at a viewing distance ranging from 50 to 75 cm (median, 60 cm). The sampling rate varied greatly because of the different eye-tracking systems used (range, 30–500 Hz; median, 60 Hz), and five studies did not report on the sampling rate. The time given to complete each viewing task was reported on in eight studies and ranged from 3 to 10 s (median, 5 s). In one study, where the stimulus material was live infants, the viewing time was not restricted, and mothers were allowed to interact freely with their infants [10]

### 3.4. Assessment Tools and Major Findings

Table 5 describes the different assessment measures and summarizes the findings for each included study. For the data analysis, each study had determined a specific AOI, which were the regions of the face or body on which the gaze patterns were to be assessed. The main AOI analyzed were the eyes, nose, and mouth. The studies identified certain measures to analyze participants’ gaze patterns. All the studies used the total fixation duration for each AOI as a primary measure to describe their findings. Other measures reported on were total fixation counts, time to first fixation, duration of first fixation, and fixation point heatmaps. Nine studies used Likert scales or questionnaires to assess the link between perceived attractiveness and the recorded visualization patterns and measures [6,7,8,10,12,18,19,20,22].

### 3.5. Assessment of Risk of Bias

The assessment results for the risk of bias are shown in Table 6. The risk of bias was moderate in eight studies, low in two studies, and high in one study.

### 3.6. Quantitative Analysis

**Figure 2:**
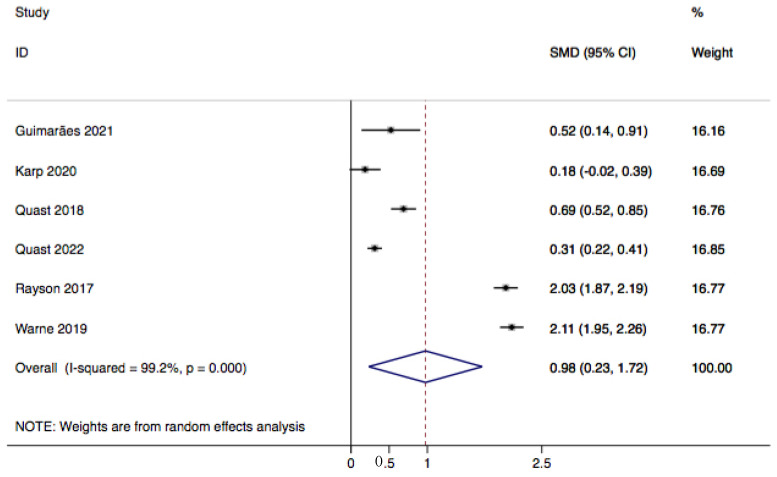
Forest plot showing standardized mean difference of the fixation duration outcome between experimental images displaying pediatric patients with CL+/−P and control images displaying pediatric patients without CL+/−P. References: [8,11,12,20,22,23].

For the fixation duration outcome, six studies were eligible for inclusion in the meta-analysis. All studies reported on fixation duration in milliseconds and reported a standard deviation. The meta-analysis results, obtained from six studies with 2776 image assessments in the experimental arm and 2288 image assessments in the control arm, showed that fixation duration differed between the experimental and control groups by 1 millisecond (standardized mean difference (SMD) 0.98, 95% CI 0.23–1.72, *p*-value = 0.01) (Figure 2).

## 4. Discussion

This systematic review is the first to comprehensively summarize the existing literature on the application of eye-tracking technology in the assessment of observers’ facial perceptions of pediatric patients with orofacial clefts. Twelve articles met the inclusion criteria; high levels in the hierarchy of evidence were not found in the study designs. Variability in the reporting study methodology was found among the studies, possibly due to the relatively new introduction of eye-tracking technology in dental research [24]. Comparatively, eye-tracking technology was introduced in the field of medicine in 1991 and its popularity peaked in 2011, 2015, and 2017 [25].

Several strengths were identified in the included studies, allowing for the reproducibility of the study settings. First, all the included studies allocated and identified AOIs, including the eyes, nose, and lips. In addition, they all reported the name of the eye-tracking machine, completed the calibration process (except for one study), and reported on the software used (except for two studies). Moreover, eye calibration prior to the experiment was completed in all the studies except for the study that used live infants [10], where the use of live stimuli hindered the applicability of the calibration process. This justified the use of images rather than live interactions in most studies.

The reproduction of the stimulus images involved digitally creating defects in normal images or enhancing the defect; mirroring the images to detract from the presence of asymmetries; and removing any facial distractors. Although the images might have closely mimicked reality, one study asked the observers whether the images looked original or digitally manipulated [21]. This indicates the considerable possibility that the images looked fake, which might have affected the study outcomes. Control stimuli were applied in 83% of the studies to compare the perceptions of the same observer to patients with or without orofacial clefts, or to steer the viewers’ knowledge of the study.

Observers were selected from different population groups. For example, one study considered mothers of children with orofacial clefts as observers [10], other studies recruited laypeople and normal children, and one study targeted adolescents with CL+/P [8]. The perceptions of these populations are of great value because they make up the social surroundings and communities in which patients with CL+/P interact in their daily lives. One study included plastic surgeons as observers [7], providing important information for guiding surgeons towards the best-practice recommendations used for repairing orofacial clefts.

Although observers’ genders were thoroughly reported on, the stimulus gender was not addressed in one third of studies. This might have affected the outcomes, as gender differences play a role in the visual perception of observers [26].

In the studies that used static images, the viewing distance was reported to be within the recommended range of 50–75 cm [14], except in two studies that did not report on this [11,20]. Fixating the viewing distance at the beginning and throughout the test time is essential to ensure that the machine can track the gaze. Another way to ensure that the observer maintains the viewing distance for proper eye movement reading is to provide a means to control head movement, which was reported on in some studies [18,21,22]. Only one study, where mothers looked at their live infants [10], reported on free-viewing.

Several parameters can be used to assess the numbers reported on by the eye-tracking software. These include fixation counts, fixation duration, first fixation duration, and time until the first fixation. Additionally, the visualization pattern can be recorded and viewed as a short video clip. In addition to the measurements retrieved from the machine, data from other assessment scales were reported on in approximately 75% of the studies. For example, the Likert scale was used to rate depression, cuteness, attractiveness, esthetics, and attention in most of the studies. The findings obtained from these assessment scales supported the machine findings and provide an understanding of how observers internally perceived stimuli, especially regarding attractiveness.

This study has limitations. First, in the NIH risk-of-bias assessment tool, four items on the checklist were given a score of zero because of the nature of the cross-sectional study design. Of the 12 included studies, nine articles had a moderate level of evidence, and two had a low risk-of-bias level. Second, there may have been heterogeneity in the included studies in the meta-analysis due to differences in the eye-tracker hardware used and in the methods of application. If the measurement tool had been unified amongst the studies, more relevant parameters could have been compared in the quantitative analysis. Lastly, although major relevant databases were searched for this systematic review, future studies should utilize other databases and include studies in other languages.

### Recommendations for Study Design and Measurements

- The inclusion and exclusion criteria for the observers and stimulus—for example, whether participants were conditioned to view syndromic children must be thoroughly reported on;
- The genders of the stimulus material must be diversified, as gender differences could play a role in observers’ perceptions;
- The time considered for fixation must be defined, and the time given to view each image should be uniform to allow for reproducibility and comparability of the reported results;
- An assessment tool specifically designed for observers’ perceptions of esthetics in CL+/P individuals is needed;
- Future studies should consider using a modified NIH risk-of-bias scoring tool to exclude irrelevant questions to the study design from the rating.

## 5. Conclusions

This systematic review and meta-analysis assessed the quality of the studies that applied eye-tracking technology in evaluating the perceptions of different populations toward pediatric patients with orofacial clefts. In this study, it was found that the methodological quality of most of the included studies was moderate. Most studies measured fixation duration and utilized a supplemental measurement scale to assess viewers’ perceptions. The meta-analysis demonstrated a significant difference in the measurements between the control groups and the patients with orofacial clefts. This might indicate the usefulness of eye-tracking technology as a metric for assessing the success of cleft repairs based on the perceptions of different populations. However, study designs, eye-tracking hardware, and eye-tracking software should be unified to allow for future comparability and reproducibility.

## Figures and Tables

**Table 1:** Search strategy on databases.

Search Terms	Database	Search Results
(Eye tracking) AND (dentistry), (Eye tracking technology) AND (dentistry), (Eye tracking) AND (Orthodontics), (Eye tracking technology) AND (Orthodontics), (Eye tracking) AND (dental surgery), (Eye tracking technology) AND (dental surgery), (Eye tracking) AND (Pediatric dentistry), (Eye tracking technology) AND (Pediatric dentistry), (Eye tracking) AND (orofacial cleft), (Eye tracking technology) AND (orofacial cleft), (Eye tracking) AND (orofacial cleft) AND ((children) OR (pediatric)), (Eye tracking technology) AND (orofacial cleft) AND ((children) OR (pediatric)), (Eye tracking) AND (cleft), (Eye tracking) AND (cleft) AND ((children) OR (pediatric)), (Eye tracking technology) AND (cleft), (Eye tracking technology) AND (cleft) AND ((children) OR (pediatric)), (Eye tracking) AND (cleft lip), (Eye tracking) AND (cleft lip) AND ((children) OR (pediatric)), (Eye tracking technology) AND (cleft lip), (Eye tracking technology) AND (cleft lip) AND ((children) OR (pediatric)), (Eye tracking) AND (cleft palate), (Eye tracking) AND (cleft palate) AND ((children) OR (pediatric)), (Eye tracking technology) AND (cleft palate), (Eye tracking technology) AND (cleft palate) AND ((children) OR (pediatric)), (Eye tracking) AND (cleft lip OR cleft palate), (Eye tracking) AND (cleft lip OR cleft palate) AND ((children) OR (pediatric)), (Eye tracking technology) AND (cleft lip OR cleft palate), (Eye tracking technology) AND (cleft lip OR cleft palate) AND ((children) OR (pediatric)).	PubMed	441
	Science Direct	6157
	Wiley	3100
	Web of Science	556

**Table 2:** Study characteristics of the included studies (n = 12).

Author(s)/Year	Country	Study Design	Inclusion Criteria	Exclusion Criteria
De Pascalis 2017 [10]	UK	Prospective Cohort	Infants with CL+/P	Infants with additional congenital disorders
Rayson 2017 [22]	UK	Cross-sectional	- Normal vision - No personal experience of an infant with a facial disfigurement	NR
Quast 2018 [12]	Germany	Cross-sectional	- Normal vision - No family history of congenital facial anomalies	NR
Boonipat 2018 [18]	US, Egypt, Thailand	Cross-sectional	20/40 vision or better is required in each eye for inclusion (lens correction permitted)	less than 20/40 vision
Morzycki 2019 [21]	Canada	Cross-sectional	- Adults 18+ years, capable of normal eye movements - Able to see at a 60 cm distance without corrective lenses	NR
Warne 2019 [23]	US	Cross-sectional	NR	NR
Kwong 2019 [7]	US	Cross-sectional	NR	NR
Karp 2020 [11]	US	Cross-sectional	- 5 to 6 years (young elementary) - 10 years (late elementary) - 13 years (middle school) - 16 years (high school) - Able to tolerate the eye-tracking glasses - Caucasian	- Non-English speaking - Developmentally delayed - Autism spectrum disorder - Impaired vision - Unsuccessful calibration
Guimarães 2021 [20]	Brazil	Cross-sectional	- Observers did not present any neurological alterations - Observers were not on medication associated with the interference of cognitive skills - Good vision	NR
Quast 2022 [8]	Germany	Cross-sectional	Normal vision	Neurological diseases or intellectual disabilities
Guimarães 2022 [19]	Brazil	Cross-sectional	- Signed informed consent - Good vision - Not taking any medication that might interfere with their cognitive or motor skills	- Neurological alterations - Recent use of drugs, alcohol, or medication that interfere with cognitive abilities - “poor” and “redo” in calibration
Hartmann 2022 [6]	Brazil	Cross-sectional	- Absence of neurological and/or visual conditions; use of alcohol, medications or drugs that could interfere with cognitive abilities, and having completed the questionnaire. - Perfect calibration.	NR

CL+/P = cleft lip and or cleft palate; NR = not reported.

**Table 3:** Observers and stimuli characteristics of the included studies (n = 12).

Author(s)/ Year	Viewed Defect	Stimuli Displaying CL+/P	Control	Observers Sample Size	Observers Gender	Stimuli Gender	Observers Age	Stimuli Age	Type of Observers
De Pascalis 2017 [10]	CL, with or without CP	10 Live Infants	20	30 mothers	30 F	10 indices with cleft (8 F, 2 M) and 20 control (7 F, 13 M)	NR	Infants at 1, 3, 5, 7, and 9 weeks postpartum	Mothers
Rayson 2017 [22]	CL	13 Images of infants with CL	13	36	18 F 18 M	NR	Mean = 25.9 y	Infants (with cleft = 2 m, control = 5.5 m)	Laypeople
Quast 2018 [12]	Unilateral CL+P	20 (×2 mirrored images)	10 (×2 mirrored images)	30	15 F, 15 M	4 F, 6 M	Mean = 25.5 y	Infants (mean = 3.3 y)	Laypeople
Boonipat 2018 [18]	Repaired CL	41 Images	95 images	403	176 F, 186 M, and 41 without specified gender	NR	18–80 y	Mean age of the F images was 11.76 y, and of M images was 13.55 y	Laypeople
Morzycki 2019 [21]	- Repaired unilateral CL with secondary deformities. - Lip scar with no secondary deformity.	15 images of different secondary unilateral CL deformities, a lip scar with no secondary deformity	4 normal Scarless lip	46	21 F, 25 M	M	18+ y	Child	Laypeople
Warne 2019 [23]	- Repaired CL - Hemifacial microsomia	- Experiment 1: 10 Repaired unilateral CL group; and 10 with hemifacial microsomia who had received no surgical treatment. - Experiment 2: 12 Repaired unilateral CL/P	Experiment 1: 10 Experiment 2: 12	Experiment 1: 11 Experiment 2: 42	Experiment 1: NR Experiment 2: 19 F 23 M	M	Experiment 1: NR Experiment 2: 21–61 y	3–15 y	Laypeople
Kwong 2019 [7]	Repaired unilateral CL by either the Fisher, Millard, or Mohler technique.	15 Unilateral CL that had been repaired by the Fisher, Millard, or Mohler technique, with 5 images provided for each technique.	5 images without deformity	30	NR	NR	NR	4–6 y	Laypeople and professionals with varying levels of experience in plastic surgery
Karp 2020 [11]	Repaired unilateral CL with secondary deformity	10 Images of unilateral CL with secondary deformity	2 images with no facial scarring	60	25 F, 35 M	NR	5–16 y	Children	Children and adolescents
Guimarães 2021 [20]	Repaired unilateral and bilateral CL	2 Images (one with unilateral and the other with bilateral lip scar)	1	40	24 F, 16 M	M	18–45 y	Adolescent	Laypeople
Quast 2022 [8]	Repaired CLP	16 Images of patients with and without CLP with neutral or smiling facial expressions.	16	54	28 F, 26 M	16 F, 16 M	10–13 y	Adolescent	Adolescents with and without CLP
Guimarães 2022 [19]	Repaired CL with secondary deformity	4 Images (one non-smiling, 3 smiling with different IOTNs)	None	91	47 F, 44 M	M	15–60+ y	Adolescent	Laypeople
Hartmann 2022 [6]	Repaired unilateral CL associated with nasal deformity	4 Images	None	85	44 F, 41 M	M	15–60+ y	Adolescent	Laypeople

CL = cleft lip; CP = cleft palate; M = male; F = female; NR = not reported; y = years; CL+P = cleft lip and palate; IOTN = Index of Orthodontic Treatment Need.

**Table 4:** Eye-tracking apparatus and application of the included studies (n = 12).

Author(s)/ Year	Eye-Tracking Machine	Eye-Tracking Software	Calibration Was Done	Viewing Distance (cm)	Control for Any Head Movement	Sampling Rate/Frequency (Hz)	Time Given per Image (sec)	Time Considered as Fixation
De Pascalis 2017 [10]	Tobii Glasses 1 (Eye-tracker glasses)	NR	NR	Free viewing	None	NR	NA	2 s
Rayson 2017 [22]	Eyelink II (Head-mounted eye tracker)	NR	Yes	57	Chin rest	500	10	40 ms
Quast 2018 [12]	SMI iView XTM RED (Screen based)	BeGaze	Yes	70	None	60	5	NR
Boonipat 2018 [18]	EyeTech TM4 (Screen based)	EyeTech’s Quick Link API	Yes	60	Chin rest	30	10	>100 ms
Morzycki 2019 [21]	EyeLink 1000 (Screen based)	EyeLink 1000	Yes	60	Chin rest	NR	3	NR
Warne 2019 [23]	SMI RED250 mobile (Screen based)	BeGaze	Yes	50–75	None	250	3	NR
Kwong 2019 [7]	Tobii Pro X2-60 (screen based)	Tobii Pro Studio	Yes	24 inch = 61 cm	NR	60	NR	Angular velocity of eye movement was below 30 degrees per second
Karp 2020 [11]	Tobii Pro Glasses 2 (Eye-tracker glasses)	Tobii Pro Lab	Yes	NR	NR	NR	5	NR
Guimarães 2021 [20]	Eye Tribe Tracker (Screen based)	OGAMA	Yes	NR	Free viewing	30	7	NR
Quast 2022 [8]	SMI Remote 250 (Screen based)	BeGaze	Yes	60	Instructed to avoid head movement	250	5	80 ms to a maximum radius of 2.02 degrees
Guimarães 2022 [19]	Eye Tribe Tracker (Screen based)	OGAMA	Yes	75	NR	NR	NR	NR
Hartmann 2022 [6]	Eye Tribe Tracker (Screen based)	OGAMA	Yes	60	None	NR	NR	NR

NR = not reported; NA = not available; ms = millisecond.

**Table 5:** Assessment methods and findings of the included studies (n = 12).

Author(s)/ Year	Areas of Interest	Assessment Measure Used in the Analyses	Other Assessment Scale	Findings
De Pascalis 2017 [10]	- General AOIs: the infant’s face, the rest of their body, and surrounding area. - Facial AOIs: eyes, mouth, and the rest of infant’s face	- % of fixation duration - % of fixation counts	- Edinburgh Postnatal Depression Scale at week 9	Mothers of infants with a cleft gazed less often at their infant’s mouth and more often on facial areas other than the eyes or mouth compared to the control group.
Rayson 2017 [22]	- Eyes - Mouth	- Total fixation duration	- Five-point rating scale of cuteness (1 = not cute, 5 = very cute).	Participants fixated significantly longer on the mouths of infants with CL, with participants looking even longer at the mouths of infants with the most severe clefts. Infants with CL were also rated as significantly less cute than unaffected infants.
Quast 2018 [12]	- Upper face: eye, forehead, cheeks - Lower face: nose, mouth, chin	- Total fixation duration	- Emotional valence questionnaire containing a 10-point Likert scale (1 = attraction, 10 = aversion) regarding overall appearance as well as appearance of the lower face.	Participants’ total fixation duration differed significantly between infants with unilateral CL+P or unilateral CL+P and NAM appliance and unaffected infants, with mean total fixation duration being the highest on the lower face in infants with unilateral CL+P and NAM appliance. Emotional valence rate given to infants with unilateral CL+P was more negative compared to that of unaffected infants. An inserted NAM appliance reduced the negative valence significantly.
Boonipat 2018 [18]	- Forehead - Periorbital - Glabellar - Infraorbital - Lateral nasal sidewall - Mid-cheek - Nasal tip, nares, and columella - Upper lip - Lower lip, chin, mandible - Ear	- Mean fixation durations - Mean fixation counts	- Attractiveness rating (Likert 1–7)	Participants’ visual attention was directed most strongly to the upper-lip AOI in cleft-repaired faces. Individuals with a personal or family history of facial deformity visually fixated more on the perioral region of faces with repaired CL. Cleft-repaired faces were rated as less attractive by an independent rater group and garnered greater visual attention by the observer group on the upper- and lower-lip AOI when compared to naive observers.
Morzycki 2019 [21]	- Eyes - Nose - Mouth - Left ear - Right ear - Scar - Entire face	- Total fixation duration	None	Participants spent more time looking at the mouth and scar region when viewing images of CL repair with secondary deformities. When comparing a normal lip to a lip with a scar without a secondary deformity, there was no significant difference in the total fixation duration at the mouth region, indicating that a successful primary lip repair does not attract observers’ attention to the mouth.
Warne 2019 [23]	- Bilateral structures: eye, cheek, temple, ear, and each hemiface. - Midline structures: forehead, nose, nostrils, mouth, central triangle, upper lip, and the superior and inferior aspects of the face.	- Total fixation duration - Time to first fixation - First fixation duration - Sequence of gaze scan path.	None	Experiment 1: The mean fixation duration in the repaired CL group was significantly longer than in the control group and the gazes were significantly longer on the area of the face around the cleft compared to the contralateral side Experiment 2: The total fixation duration and first fixation duration in the upper-lip region was significantly longer in the CL group than in the control group. As the severity (Asher-McCade Aesthetic Index) of residual cleft deformity increased, participants fixated earlier and for longer on the CL group than on the control group.
Kwong 2019 [7]	Control images - Left and right nose - Philtrum - Left and right upper lateral subunits of the lips - Left and right sides of the vermillion. Cleft images (in addition to the above) - Scar region	- Total fixation duration - Fixation counts	- Esthetic quality of images on a 1–10 Likert scale (1, poor outcome; 10, excellent outcome)	Laypersons spent a greater proportion of fixation duration and fixation count analyzing surgical scars than any other group. Craniofacial surgeons spent the least amount of time of any group analyzing surgical scars and the most amount of time analyzing contralateral features. Fisher repairs had a significantly lower proportion of fixations counts in AOIs and were rated as the most aesthetically pleasing compared with Mohler and Millard repairs.
Karp 2020 [11]	- Mouth - Nose - Scar - Side of the mouth with a scar	- Total fixation duration - Fixation counts	None	All children spent longer fixation durations on images with CL with a secondary deformity compared with the control images. More individual gaze counts and greater amounts of time were spent looking at the mouths of SCLD images than the control images. Younger age groups spent less time looking at specific areas of interest in SCLD images.
Guimarães 2021 [20]	- Right eye - Left eye - Right nose area - Left nose area - Lower third of the face	- Total fixation duration - Fixation count - Visualization pattern - Time to first fixation, - Fixation point heatmaps	- Visual analog scale of attractiveness questionnaire. ranging from 0 = less attractive, to 10 = more attractive images	The image of bilateral CLP was primordial and strongly captured. The nose area was secondary to the areas of the lips and eyes in images without fissures. In bilateral CLP, observers fixed their attention more frequently on the upper lip than on the eyes when shown faces at rest. In images without scarring, the capture was in the eye area and not on the upper lip. Images without scars scored higher attractiveness grades.
Quast 2022 [8]	- Eyes - Nose - Mouth	- Total fixation duration - Initial attentional capture	- Attractiveness: 1 = very attractive; nine = not attractive at all. - Valence of the facial expression: 1 = very negative: five = neutral; nine = very positive.	Shorter fixations on the eyes and longer fixations on the nose and mouth of adolescents with CLP compared to their unaffected peers. Adolescents with CLP tended to spend less time fixating on the eyes. In the attractiveness/valence ratings, CLP adolescents were rated more negatively. Smiling altered the scan path toward the mouth for all faces and the valence was rated higher compared to neutral faces.
Guimarães 2022 [19]	- Eyes - Right nose - Left nose - Upper lip - Teeth with lower lip	- Total fixation duration - Time to first fixation - Visualization pattern - Fixation point heatmaps	- Visual analog scale of attractiveness of 0 = complete disagreement, to 100 = complete agreement.	Mouths and teeth had greater fixation durations regardless of the grade of IOTN. There were significant differences in the time until the first fixation on the scar of the repaired CL region for IOTN grade 1. The presence of a CL scar on the upper lip did not attract the eyes of laypeople observers of different ages, regardless of the degree of malocclusion in the non-smile image. IOTN grade 1 repaired CL regions received the highest VAS scores. The older the age, the greater the tendency to give a higher VAS score for the same malocclusion. Older observers gave higher scores than younger ones. As the severity of the malocclusion increased, they were found to be less attractive.
Hartmann 2022 [6]	- Frontal view: eyes, right side of the nose, left side of the nose, upper right lip, upper left lip, mouth - Oblique view: eyes, nose, upper right lip, upper left lip, mouth	- Total fixation duration - Time to first fixation - Fixation point heatmaps	- Six attention questionnaires that evaluated athletic performance, popularity, leadership, capability, happiness and social aspects. - Visual Analog Scale—0 = less attractive to 100 = more attractive	Greater gaze concentration was observed for the left nostril and scar, followed by the right eye and, finally, the left eye. The scar area attracted attention during the primary glance. Visible scars were associated with lower attractiveness (VAS), happiness, and intelligence, as well as shyness and sympathy; however, it did not influence the characteristic of “good hygienic habits”.

AOIs = areas of interests; CL = cleft lip, NAM = nasoalveolar molding; CL + P = cleft lip and palate; IOTN = Index of orthodontic treatment need; VAS = Visual analogue scale.

**Table 6:** Assessment of studies’ quality using the NIH quality assessment tool for observational cohort and cross-sectional studies for the included articles (n = 12).

	De Pascalis 2017 [10]	Rayson 2017 [22]	Quast 2018 [12]	Boonipat 2018 [18]	Morzycki 2019 [21]	Warne 2019 [23]	Kwong 2019 [7]	Karp 2020 [11]	Guimarães 2021 [20]	Quast 2022 [8]	Guimarães 2022 [19]	Hartmann 2022 [6]
**Q1**	**✓**	**✓**	**✓**	**✓**	**✓**	**✓**	**✓**	**✓**	**✓**	**✓**	**✓**	**✓**
**Q2**	**✓**	**✓**	**✓**	**✓**	**✓**	❌	❌	**✓**	**✓**	**✓**	**✓**	**✓**
**Q3**	**✓**	**✓**	CD, NA, NR	CD, NA, NR	CD, NA, NR	CD, NA, NR	CD, NA, NR	**✓**	CD, NA, NR	CD, NA, NR	**✓**	**✓**
**Q4**	❌	**✓**	**✓**	**✓**	**✓**	❌	❌	**✓**	**✓**	**✓**	**✓**	**✓**
**Q5**	**✓**	**✓**	❌	❌	❌	❌	❌	❌	❌	❌	**✓**	**✓**
**Q6**	**✓**	❌	❌	❌	❌	❌	❌	❌	❌	❌	❌	❌
**Q7**	**✓**	❌	❌	❌	❌	❌	❌	❌	❌	❌	❌	❌
**Q8**	**✓**	**✓**	❌	**✓**	**✓**	**✓**	**✓**	**✓**	**✓**	❌	**✓**	❌
**Q9**	**✓**	**✓**	**✓**	❌	**✓**	**✓**	**✓**	**✓**	**✓**	**✓**	**✓**	**✓**
**Q10**	**✓**	❌	❌	❌	❌	❌	❌	❌	❌	❌	❌	❌
**Q11**	**✓**	**✓**	**✓**	**✓**	**✓**	**✓**	**✓**	**✓**	**✓**	**✓**	**✓**	❌
**Q12**	CD, NA, NR	CD, NA, NR	CD, NA, NR	CD, NA, NR	CD, NA, NR	CD, NA, NR	CD, NA, NR	CD, NA, NR	CD, NA, NR	CD, NA, NR	CD, NA, NR	CD, NA, NR
**Q13**	**✓**	❌	❌	❌	❌	❌	❌	❌	CD, NA, NR	❌	❌	❌
**Q14**	**✓**	**✓**	**✓**	**✓**	❌	❌	**✓**	**✓**	❌	**✓**	❌	**✓**
**Total**	**12**	**9**	**6**	**6**	**6**	**4**	**5**	**8**	**6**	**6**	**8**	**7**
**Quality level**	**High**	**Moderate**	**Moderate**	**Moderate**	**Moderate**	**Low**	**Low**	**Moderate**	**Moderate**	**Moderate**	**Moderate**	**Moderate**

CD, NA, NR = cannot determine, not applicable, not reported.

## Data Availability

The data presented in this study are available on request from the corresponding author.

## Appendix A. NIH Guidance for Assessing the Quality of Observational Cohort and Cross-Sectional Studies

**Criteria**	**Yes**	**No**	**Other** **(CD, NR, NA) ***
1. Was the research question or objective in this paper clearly stated?			
2. Was the study population clearly specified and defined?			
3. Was the participation rate of eligible persons at least 50%?			
4. Were all the subjects selected or recruited from the same or similar populations (including the same time period)? Were inclusion and exclusion criteria for being in the study prespecified and applied uniformly to all participants?			
5. Was a sample size justification, power description, or variance and effect estimates provided?			
6. For the analyses in this paper, were the exposure(s) of interest measured prior to the outcome(s) being measured?			
7. Was the timeframe sufficient so that one could reasonably expect to see an association between exposure and outcome if it existed?			
8. For exposures that can vary in amount or level, did the study examine different levels of the exposure as related to the outcome (e.g., categories of exposure, or exposure measured as continuous variable)?			
9. Were the exposure measures (independent variables) clearly defined, valid, reliable, and implemented consistently across all study participants?			
10. Was the exposure(s) assessed more than once over time?			
11. Were the outcome measures (dependent variables) clearly defined, valid, reliable, and implemented consistently across all study participants?			
12. Were the outcome assessors blinded to the exposure status of participants?			
13. Was loss to follow-up after baseline 20% or less?			
14. Were key potential confounding variables measured and adjusted statistically for their impact on the relationship between exposure(s) and outcome(s)?			
* CD, NA, NR = cannot determine, not applicable, not reported.			

## Appendix B. Quantitative Analysis of the Included Studies (n = 6)

**Author(s)/Year**	**No. of Participants**	**No. of Experiment Images**	**No. of Control Images**	**Fixation Duration Experiment**		**Fixation Duration** **Control**	
				**Value**	**SD**	**Value**	**SD**
Guimarães 2021 [20]	40	80	40	885	906	488	294
Karp 2020 [11]	56	560	112	4230	1410	3970	1420
Quast 2018 [12]	30	300	300	2499	571	2129	502
Quast 2022 [8]	54	864	864	283	205	224	174
Rayson 2017 [22]	36	468	468	3069	820	1542	679
Warne 2019 [23]	42	504	504	846	135	574	123
